# Arbeitsunfähigkeit bei präklinischen Rettungskräften in Deutschland: Ergebnisse der EMS-Health-Studie

**DOI:** 10.1007/s40664-023-00497-x

**Published:** 2023-03-07

**Authors:** Tobias May, Christina Arnold, Teresa Klas, Christina Möckel, Leona Maaß, Thomas Hofmann, Luis Möckel

**Affiliations:** 1 HSD Hochschule Döpfer GmbH, University of Applied Sciences, Köln, Deutschland; 2 Hochschule Fresenius, University of Applied Sciences, Köln, Deutschland; 3 Forschungszentrum Jülich, Jülich, Deutschland; 4 Fachbereich für Human- und Gesundheitswissenschaften, Universität Bremen, Bremen, Deutschland; 5 IU Internationale Hochschule GmbH, University of Applied Sciences, Hildebrandtstraße 24c, 40215 Düsseldorf, Deutschland

**Keywords:** Krankmeldung, Präklinische Einsatzkräfte, Chronische Krankheiten, Rettungsdienst, Gesundheit, Notification of sickness, Prehospital emergency medical services staff, Chronic diseases, Rescue service, Health

## Abstract

**Hintergrund:**

Für Krankenstandanalysen ist die Ermittlung von Arbeitsunfähigkeit ein zentraler Ansatz. Dennoch liegen für Arbeitsunfähigkeit und damit assoziierte Faktoren im deutschen präklinischen Rettungsdienst bisher noch keine Daten vor.

**Ziel der Arbeit:**

Ziel dieser Analyse war es, den Anteil der Rettungskräfte in Deutschland mit mindestens einer Arbeitsunfähigkeit in den letzten 12 Monaten und damit assoziierte Faktoren zu identifizieren.

**Material und Methoden:**

Es handelt sich um eine bundesweite Befragungsstudie mit Rettungskräften. Assoziationen zwischen der Arbeitsunfähigkeit und soziodemografischen, gesundheitsbezogenen sowie berufsspezifischen Faktoren wurde mittels multivariabler logistischer Regression, unter Berechnung von Odds-Ratio (OR) und dazugehörigen 95 % Konfidenzintervallen (95 % KI) identifiziert.

**Ergebnisse:**

In diese Analyse eingeschlossen wurden 2298 deutsche Rettungskräfte. 60,10 % der weiblichen sowie 58,98 % der männlichen Befragten gaben mindestens eine Arbeitsunfähigkeit in den letzten 12 Monaten an. Eine Arbeitsunfähigkeit war unter anderem signifikant mit dem Schulabschluss (Abitur: OR: 0,51 [95 % KI 0,30–0,88]; *p* = 0,016; Referenz: Hauptschulabschluss) und den wöchentlichen Arbeitsstunden (OR: 1,01 [95 % KI 1,00–1,02]; *p* = 0,003) assoziiert. Auch das Arbeitsumfeld, die Dienstjahre sowie verschiedene physische und psychische Beschwerden in den letzten 12 Monaten zeigten eine signifikante Assoziation mit einer Arbeitsunfähigkeit.

**Diskussion:**

Diese Analyse weist darauf hin, dass unter anderem chronische Krankheiten, der Bildungsabschluss, das Einsatzgebiet sowie die Anzahl der Dienstjahre und wöchentlichen Arbeitsstunden bei den teilnehmenden Rettungskräften mit Arbeitsunfähigkeitstagen in den letzten 12 Monaten assoziiert waren.

## Abstract (en)

**Background:**

The determination of incapacity to work is a central approach for analyses of absence due to sickness. Nevertheless, no data are yet available for incapacity to work and associated factors in the German prehospital emergency medical services (EMS) staff.

**Objective:**

The aim of this analysis was to identify the proportion of EMS staff with at least one incapacity for work (AU) in the previous 12 months and associated factors.

**Material and methods:**

This was a nationwide survey study with rescue workers. Factors associated with work disability were identified using multivariable logistic regression, calculating odds ratios (OR) and associated 95% confidence intervals (95% CI).

**Results:**

Included in this analysis were 2298 employees of the German emergency medical services (female 42.6%, male 57.2%). Overall, 60.10% of female participants and 58.98% of male participants reported an incapacity for work in the previous 12 months. Incapacity for work was significantly associated with having a high school diploma (high school diploma: OR: 0.51, 95% CI 0.30; 0.88, *p* = 0.016; reference: secondary school diploma), working in a rural environment (OR: 0.65, 95% CI 0.50; 0.86, *p* = 0.003) or urban environment (OR: 0.72, 95% CI: 0.53; 0.98, *p* = 0.037). Furthermore, hours worked per week (OR: 1.01, 95% CI: 1.00; 1.02, *p* = 0.003) and 5–< 10 years of service (OR: 1.40, 95% CI: 1.04; 1.89, *p* = 0.025) were associated with higher odds of work disability. Neck and back pain, depression, osteoarthritis, and asthma in the previous 12 months also showed a significant association with work disability in the same time period.

**Conclusion:**

This analysis shows that chronic diseases, educational attainment, area of assignment, years of service, and hours worked per week, among others, were associated with incapacity for work in the previous 12 months in German EMS staff.

Im deutschen Rettungsdienst sind ca. 72.000 Beschäftigte tätig [26]. Die Mitarbeitenden auf den Einsatzmitteln erfüllen verschiedene Qualifikationen. Notfallsanitäter*innen haben die höchste nichtärztliche Qualifikation im Rettungsdienst. Das dazugehörige Berufsgesetz ist am 01.01.2014 in Kraft getreten und löst das Berufsbild des Rettungsassistenten/der Rettungsassistentin, als bis dahin höchste rettungsdienstliche Qualifikation, ab. Die Bezeichnung des Rettungssanitäters/der Rettungssanitäterin erfüllt die Mindestanforderung um im Rettungsdienst tätig zu sein. Rettungshelfer*innen werden im Krankentransport eingesetzt. Zudem müssen notarztbesetze Rettungsmittel mit einem Arzt besetzt werden, der die Zusatzbezeichnung Notfallmedizin erworben hat [27]. Die sozioepidemiologische und demografische Entwicklung der Bevölkerung sowie allgemeine Veränderungen im Gesundheitswesen haben Auswirkungen auf den deutschen präklinischen Rettungsdienst. Der Umfang der Versorgung durch die Rettungskräfte verändert sich und neue Notfallversorgungssysteme, wie beispielsweise Intensivtransport- und/oder Schwerlastrettungswagen müssen besetzt werden, um den aktuellen präklinischen Anforderungen gerecht zu werden [20]. Das Einsatzaufkommen hat sich von 1994 bis 2013 fast verdoppelt und steigt jährlich um etwa 5 % an [20, 22], was tendenziell zu einer steigenden physischen und psychischen Belastung der Rettungskräfte führen kann. Die Mitarbeitenden sind ständig wechselnden Gegebenheiten, hoher Verantwortung und körperlichen Herausforderungen ausgesetzt. Hinzu kommen lange Arbeitszeiten und Wechselschichten an 365 Tagen im Jahr. Die durchschnittliche wöchentliche Arbeitszeit aller Erwerbstätigen in Deutschland betrug 2019 34,8 h [2], im Rettungsdienst hingegen können 48 Arbeitsstunden pro Woche die Regel sein [15]. Das Personal ist zudem Gewalterfahrungen durch Patient*innen und Angehörige ausgesetzt [14, 19, 21]. Diese Gewalterfahrungen könnten wiederum Auswirkungen auf den körperlichen und psychischen Gesundheitszustand der Rettungskräfte haben [8, 14].

Möckel et al. (2021) konnten in einer Querschnittstudie zeigen, dass 58,64 % der teilnehmenden Rettungskräfte unter Schmerzen litten [12]. Des Weiteren waren Schmerzen bei Rettungskräften signifikant mit den Jahren im Rettungsdienst sowie dem Geschlecht assoziiert [13]. Aber auch für Depressionen, Allergien und Herz-Kreislauf-Erkrankungen konnte in der *EMS-Health-Studie*, welche auch die Grundlage für die vorliegende Arbeit bildet, eine hohe Prävalenz unter Rettungskräften identifiziert werden [11]. Die Mitarbeitenden im Rettungsdienst gelten als anfällig für Burnout-Erkrankungen, welche in Zusammenhang mit dem Wohlbefinden, der körperlichen Belastung und der Arbeitszufriedenheit stehen [1, 4, 9].

Basierend auf den Arbeitsunfähigkeitsmeldungen der 14,1 Mio. erwerbstätigen AOK-Mitglieder in Deutschland waren muskuloskeletale (22,1 %) und psychische (12,0 %) Beschwerden 2020 die häufigsten Gründe für Arbeitsunfähigkeitstage (AU-Tage) bei den Versicherten [10]. Außerdem konnte bereits für die Alten- und Krankenpflege, welche stellenweise vergleichbare berufliche Merkmale aufweisen wie die Beschäftigung im Rettungsdienst, ein deutlich höherer Krankheitsausfall im Vergleich zur Gesamtgruppe der Berufstätigen gezeigt werden [25]. Daten von weiteren Berufsgruppen, die einen beruflichen Vergleich zum Rettungsdienst zulassen, können den oben beschriebenen Beschwerden gegenübergestellt werden. So konnten Kleiber et al. bei einer Befragung von deutschen Polizeibeamt*innen (*n* = 941) identifizieren, dass die häufigsten Prävalenzen von gesundheitlichen Beschwerden in den letzten 12 Monaten Schlafstörungen sowie Beschwerden im Rücken- und Nackenbereich waren. Des Weiteren korrelierte die emotionale Erschöpfung mit einer Arbeitsunfähigkeit bei den Polizist*innen [28].

Für den deutschen Rettungsdienst liegen bisher keine Daten vor, welche die Häufigkeit von Arbeitsunfähigkeit (AU) sowie damit assoziierte Faktoren untersuchen. Somit war das Ziel dieser Analyse, den Anteil der teilnehmenden Rettungskräfte mit mindestens einer AU in den letzten 12 Monaten zu bestimmen sowie mit dieser AU assoziierte Faktoren zu identifizieren.

## Methode

### Studiendesign und Untersuchungsmethoden

Bei der *EMS-Health-Studie, *in welcher die Daten für die vorliegende Analyse gesammelt wurden, handelte es sich um eine bundesweite Querschnittstudie mit deutschen Rettungskräften, die als Onlinebefragung zwischen dem 19. Mai und 1. Juni 2021 durchgeführt wurde. Der Link zur Onlinebefragung wurde über die Deutsche Gesellschaft für Rettungswissenschaften (DGRe), Rettungsdienst-Online-Gruppen und durch die Bereitstellung des Links an 332 Rettungsdienststationen verteilt. Die Anzahl der angeschriebenen Rettungswachen je Bundesland wurde basierend auf dem Bevölkerungsanteil des jeweiligen Bundeslandes an der Deutschen Gesamtbevölkerung getroffen. Die Rettungswachen selbst wurden durch eine Internetrecherche identifiziert und per E‑Mail angeschrieben, mit der Bitte, den Link zur Onlinebefragung an die präklinischen Rettungskräfte weiterzuleiten. Bei der Auswahl der Rettungswachen wurde nicht unterschieden, ob es sich bei diesen um Berufsfeuerwehren, Hilfsorganisationen oder kommunale Trägerschaften handelte.

Die Studie wurde unter Beachtung der Deklaration von Helsinki in der aktuellen Fassung durchgeführt und die folgenden Schritte zur Einhaltung unternommen. Zunächst wurden die Teilnehmenden vorab in einem Einleitungstext über Ziele, Methoden und den potenziellen Nutzen der Studie informiert. Die Studie war anonym, freiwillig und die Teilnehmenden gaben ihre informierte Einwilligung zur Teilnahme an der Studie. Teilnehmende, welche ihre Einwilligung nicht erteilten, wurden nicht in die eigentliche Befragung geleitet und haben mit Ausnahmen des Einleitungstextes und des Einwilligungsitems, kein weiteres Item der Befragung beantworten können. Zum Schutz der Teilnehmenden konnte die Befragung jederzeit durch die Teilnehmenden beendet werden, sollte bei diesen bspw. Unwohlsein in Bezug auf gestellte Fragen aufgekommen sein. Da auch nach dem Vorliegen einer Depression gefragt wurde, lag ein Hinweis vor, wo man sich entsprechende Hilfe suchen könnte. Außerdem wurden die aktuell geltenden Datenschutzrichtlinien bei der Erhebung sowie der Bearbeitung der Daten eingehalten. Entsprechend der Deklaration von Helsinki lag ein Ethikvotum der Ethikkommission der HSD Hochschule Döpfer (Köln) für die Durchführung dieser Studie vor (Antragszeichen: BEth_M05_2021).

### Fragebogen

Für die Datenerhebung wurden soziodemografische, berufs- sowie gesundheitsbezogene Merkmale erhoben. Während die berufsbezogenen Items selbst entwickelt waren, wurden die gesundheitsbezogenen Items dem Fragebogen des Robert-Koch-Instituts zur Studie „Gesundheit in Deutschland aktuell“ (GEDA) entnommen, wobei zum Zeitpunkt der Erhebung der aktuellste zur Verfügung stehende Fragebogen der zur GEDA2014/2015-Studie war [18]. Als gesundheitsbezogene Daten wurden Größe und Gewicht der Teilnehmenden abgefragt, ob eine chronische Krankheit vorliegt (definiert als *Krankheiten oder gesundheitliche Probleme, die mindestens 6 Monate andauern oder voraussichtlich andauern werden*) sowie ob in den letzten 12 Monaten die folgenden Krankheiten/Beschwerden vorlagen: Rückenschmerzen, Diabetes, COPD, Allergien (ohne allergisches Asthma), Hypertonie, Depression, erhöhte Blutfette [11, 18].

Zur Erhebung der Arbeitsunfähigkeit in den letzten 12 Monaten wurde die folgende Frage gestellt, „*Wenn Sie erwerbstätig sind, kam es in den letzten 12 Monaten vor, dass Sie krankheitsbedingt nicht zur Arbeit gehen konnten?*“ (Antwortoptionen: Ja/Nein), wobei der folgende Hinweis an die Studienteilnehmenden gegeben wurde: *Bitte berücksichtigen Sie alle Krankheiten, Verletzungen und sonstige gesundheitliche Beschwerden, wegen derer Sie nicht arbeiten konnten*. Teilnehmende, welche angaben, in den letzten 12 Monaten arbeitsunfähig gewesen zu sein, wurden nach der Anzahl der Tage gefragt („*Wie viele Tage haben Sie in den letzten 12 Monaten insgesamt krankheitsbedingt bei der Arbeit gefehlt?*“) [18]. Weitere Angaben zum Studiendesign und zum Fragebogen sind in Möckel et al. (2022) dargestellt [11].

### Datenanalyse

In die vorliegende Analyse eingeschlossen wurden alle Teilnehmenden, die mindestens 18 Jahre alt waren, aktiv in der präklinischen Notfallrettung seit mindestens einem Jahr tätig waren und die Hauptfrage „*Wenn Sie erwerbstätig sind, kam es in den letzten 12 Monaten vor, dass Sie krankheitsbedingt nicht zur Arbeit gehen konnten?*“ beantwortet haben, wobei eine der Antwortoptionen (Ja/Nein) angekreuzt werden musste.

Die statistische Auswertung wurde mit dem JASP-Software-Paket in der Version 0.16 (Amsterdam, Niederlande) durchgeführt [7]. Der Anteil der Studienteilnehmenden mit mindestens einer AU in den letzten 12 Monaten wurde für die Gesamtstudienpopulation nach Geschlecht sowie nach Altersgruppe berechnet. Des Weiteren wurde die durchschnittliche Anzahl an AU-Tagen sowie der dazugehörige Standardfehler bei Teilnehmenden mit einer AU in den letzten 12 Monaten sowie der Anteil der Teilnehmenden mit > 30 AU-Tagen berechnet.

Um Assoziationen zwischen soziodemografischen (u. a. Geschlecht, Alter, Schulabschluss, Familienstand), berufs- (u. a. Schichtdienst, Einkommen, Arbeitsumfeld) sowie gesundheitsbezogenen (u. a. Rauchverhalten, BMI, Vorliegen verschiedener Erkrankungen in den letzten 12 Monaten) Faktoren und einer Arbeitsunfähigkeit in den letzten 12 Monaten zu ermitteln, wurden zunächst univariable Analysen mittels Chi-Quadrat-Test (χ^*2*^) bzw. logistischer Regression (Wald Statistik) durchgeführt. Um Confouding-Effekte zu minimieren, wurden Faktoren, welche in den univariablen Analysen einen *p* < 0,1 zeigten sowie in jedem Fall das Geschlecht, anschließend in einer multivariablen logistischen Regression unter Berechnung von adjustierten Odds-Ratios (OR) und dazugehörigen 95 % Konfidenzintervalle (95 % KI) analysiert. Subgruppen mit *n* < 10 Teilnehmenden wurden nicht separat in der multivariablen Analyse berücksichtigt. Ein *p*-Wert von ≤ 0,05 wurde als statistisch signifikant betrachtet.

## Ergebnisse

### Charakteristika der Teilnehmenden

Insgesamt wurden 2298 Rettungskräfte aus der *EMS-Health-Studie* in die vorliegende Analyse eingeschlossen. Von diesen hatten 1367 teilnehmende Rettungskräfte mindestens eine AU in den letzten 12 Monaten, wohingegen 931 nicht von einer Arbeitsunfähigkeit betroffen waren (Tab. 1). Die Verteilung nach Geschlecht war in der Gruppe ohne bzw. mit AU vergleichbar, wobei 42,0 % bzw. 43,1 % weiblich und 57,9 % bzw. 56,7 % männlich waren. In der Gruppe mit AU war der Anteil der 18- bis 29-Jährigen (61,0 %) niedriger als in der ohne AU (71,5 %), dagegen waren anteilig mehr Teilnehmende zwischen 30 und 44 Jahren in der AU-Gruppe (27,3 %) im Vergleich zur nicht AU-Gruppe (19,7 %). Eine leicht unterschiedliche Verteilung war auch für den Familienstand zu beobachten mit 71,9 % bzw. 81,3 % Singles und 27,7 % bzw. 18,5 % verheiratet/in Partnerschaft lebend in der AU- und nicht-AU-Gruppe (Tab. 1). Bei den Teilnehmenden mit AU in den letzten 12 Monaten waren am häufigsten die Notfallsanitäter*innen (43,0 %) vertreten, wohingegen in der Gruppe ohne AU die Rettungssanitäter*innen (40,1 %) die häufigste Berufsgruppe stellten. Raucher waren in beiden Gruppen mit 26,0 % (AU) und 24,9 % (ohne AU) vergleichbar häufig repräsentiert. Alle Details zu den Charakteristika der Studienteilnehmenden sind in Tab. 1 dargestellt.

**Table 1:** 

	*Mit* AU in den letzten 12 Monaten (in %) *(n* *=* *1367)*	*Ohne* AU in den letzten 12 Monaten (in %) *(n* *=* *931)*
*Geschlecht*		
Weiblich	43,1	42,0
Männlich	56,7	57,9
Divers	0,2	0,1
*Raucher*		
Nein, noch nie	53,2	59,4
Nein, nicht mehr	20,7	15,7
Ja	26,0	24,9
*Familienstatus*		
Single	71,9	81,3
Verheiratet/in Lebenspartnerschaft	27,7	18,5
Verwitwet/Partner verstorben	0,3	0,1
*Beruf*		
Notfallsanitäter*in	43,0	30,9
Rettungsassistent*in	6,2	5,7
Rettungssanitäter*in	33,2	40,1
Rettungshelfer*in	2,9	3,9
Notärzt*in	1,3	0,9
Auszubildende	13,3	18,6
*Altersgruppen*		
18–29 Jahre	61,0	71,5
30–44 Jahre	27,3	19,7
45–64 Jahre	11,7	8,8

### Arbeitsunfähigkeit bei teilnehmenden Rettungskräften

**Figure 1:**
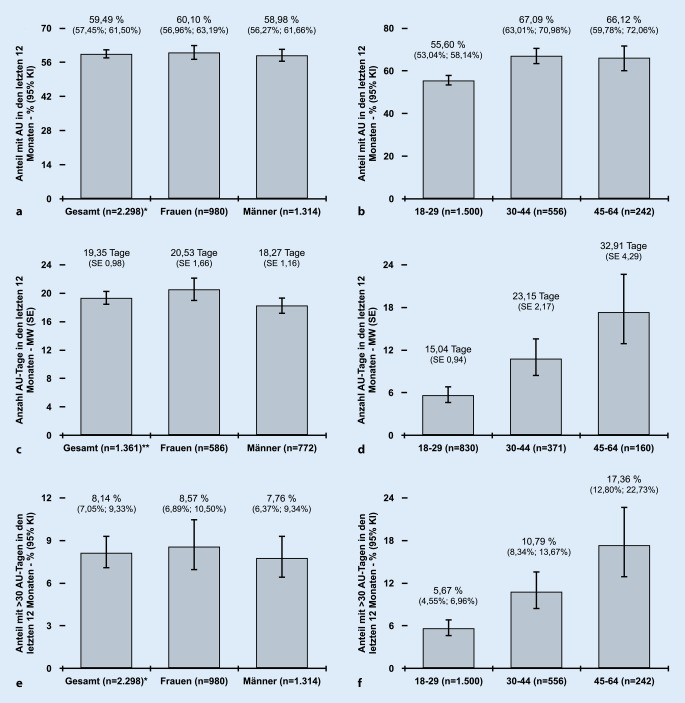

Insgesamt gaben 59,49 % (95 % KI 57,45–61,50) der Studienteilnehmenden an, in den letzten 12 Monaten mindestens eine AU gehabt zu haben (Abb. 1a). Dabei waren Frauen mit 60,10 % (95 % KI 56,96–63,19) etwa vergleichbar häufig betroffen wie männliche Studienteilnehmende mit 58,98 % (95 % KI 56,27–61,66). Nach Altersgruppe betrachtet, war bei den 18- bis 29-Jährigen (55,60 % [95 % KI 53,04–58,14]) der Anteil mit einer AU in den letzten 12 Monaten am geringsten, wohingegen bei den 30- bis 44-Jährigen (67,09 % [95 % KI 63,01–70,98]) der Anteil am höchsten war (Abb. 1b). Mehr als 30 AU-Tage in den letzten 12 Monaten nahmen 8,14 % (95 % KI 7,05–9,33) der Studienteilnehmenden (Abb. 1e). Dabei war der Anteil von Teilnehmenden mit über 30 AU-Tagen bei den 18- bis 29-Jährigen (5,67 % [95 % KI 4,55–6,96]) geringer als bei den 30- bis 44-Jährigen (10,79 % [95 % KI 8,34–13,67]) und den 45- bis 64-Jährigen (17,36 % [95 % KI 12,80–22,73]; Abb. 1f).

Die Anzahl der AU-Tage in den letzten 12 Monaten lag für die Gesamtstichprobe (*n* = 2298) bei 11,38 (SE 0,61) sowie für die Gruppe der Teilnehmenden mit mindestens einer AU in den letzten 12 Monaten (*n* = 1361) bei 19,35 (SE 0,98) (Abb. 1c). Die folgenden Zahlen beziehen sich ausschließlich auf die Gruppe der Teilnehmenden mit mindestens einer AU in den letzten 12 Monaten. So war ersichtlich, dass Studienteilnehmerinnen mit 20,53 (SE 1,66) numerisch mehr AU-Tage angaben als Teilnehmer mit 18,27 (SE 1,16) AU-Tagen (Abb. 1c). Deutlich zu erkennen ist, dass mit Anstieg des Alters auch die Anzahl der AU-Tage zunimmt, wobei die 45- bis 64-Jährigen im Mittel 32,91 (SE 4,29) AU-Tage und die 18- bis 29-Jährigen 15,04 (SE 0,94) AU-Tage in den letzten 12 Monaten nehmen mussten (Abb. 1d).

### Mit Arbeitsunfähigkeit assoziierte Faktoren

In der multivariablen logistischen Regression signifikant mit einer geringeren Chance für eine AU in den letzten 12 Monaten assoziiert, waren der Schulabschluss Abitur (OR: 0,51 [95 % KI 0,30–0,88]; *p* = 0,016) im Vergleich zur Referenzgruppe Haupt/-Volkschulabschluss (Tab. 2) sowie die Arbeit in einem ländlichen (OR: 0,65 [95 % KI 0,50–0,86]; *p* = 0,003) bzw. städtischen Umfeld (OR: 0,72 [95 % KI 0,53–0,98]; *p* = 0,037) im Vergleich zur Arbeit in der Großstadt.

**Table 2:** 

Merkmale	Univariable Analyse *χ*^*2*^/Wald; *p*-Wert	Multivariable Analyse^a^ OR (95 % KI); *p*-Wert
*Geschlecht*		
Weiblich (*n* = 980)	0,693; *p* = 0,707	Referenz
Männlich (*n* = 1314)		1,04 (0,49; 2,21); *p* = 0,93
Divers (*n* = 4)^b^		–
*Altersgruppen*		
18–29 Jahre (*n* = 1500)	27,14; *p* ≤ 0,001	Referenz
30–44 Jahre (*n* = 556)		0,90 (0,63; 1,28); *p* = 0,565
45–64 Jahre (*n* = 242)		0,81 (0,46; 1,43); *p* = 0,468
*Schulabschluss*		
Haupt‑/Volkschulabschluss (*n* = 108)	25,13; *p* ≤ 0,001	Referenz
Mittlere Reife (*n* = 679)		0,59 (0,35; 1,01); *p* = 0,055
Fachhochschulreife (*n* = 356)		0,64 (0,36; 1,13); *p* = 0,125
Abitur (*n* = 1154)		*0,51 (0,30; 0,88); p* *=* *0,016*
Abschluss nach 7 Jahren (*n* = 1)^b^		–
*Familienstand*		
Ledig (*n* = 1740)	26,82; *p* ≤ 0,001	Referenz
Verheiratet (*n* = 550)		1,28 (0,95; 1,73); *p* = 0,103
Verwitwet/Partner verstorben (*n* = 5)^b^		–
*Nettoeinkommen*		
< 1000 € (*n* = 189)	40,84; *p* ≤ 0,001	0,71 (0,47; 1,07); *p* = 0,105
1000 bis < 2000 (*n* = 451)		1,00 (0,74; 1,34); *p* = 0,977
2000 bis < 3000 (*n* = 684)		Referenz
3000 bis < 4000 (*n* = 358)		1,24 (0,92; 1,67); *p* = 0,159
4000 bis < 5000 (*n* = 262)		1,04 (0,74; 1,45); *p* = 0,825
> 5000 (*n* = 176)		0,98 (0,65; 1,46); *p* = 0,906
*Schichtdienst*		
Nein (*n* = 200)	1,65; *p* = 0,2	–
Ja (*n* = 1946)		
*Tatsächliche Stunden wöchentliche Arbeitszeit – pro Stunde Anstieg*	27,72; *p* ≤ 0,001	*1,01 (1,00; 1,02); p* *=* *0,003*
*Arbeitsumgebung*		
Großstadt (*n* = 489)	8,37; *p* = 0,039	Referenz
Städtische Umgebung (*n* = 343)		*0,72 (0,53; 0,98); p* *=* *0,037*
Ländliche Umgebung (*n* = 575)		*0,65 (0,50; 0,86); p* *=* *0,003*
Städtische als auch ländliche (*n* = 743)		0,84 (0,65; 1,10); *p* = 0,209
*Beruf*		
Notfallsanitäter*in (*n* = 876)	41,14; *p* ≤ 0,001	Referenz
Rettungsassistent*in (*n* = 138)		0,82 (0,53; 1,27); *p* = 0,379
Rettungssanitäter*in (*n* = 827)		0,90 (0,67; 1,22); *p* = 0,51
Rettungshelfer*in (*n* = 76)		1,50 (0,78; 2,90); *p* = 0,299
Notärztin/Notarzt (*n* = 26)		1,41 (0,52; 3,82); *p* = 0,498
In Ausbildung (*n* = 355)		0,94 (0,64; 1,36); *p* = 0,727
*Rauchverhalten*		
Noch nie (*n* = 1279)	11,64; *p* = 0,003	Referenz
Nicht mehr (*n* = 429)		1,06 (0,81; 1,39); *p* = 0,661
Ja (*n* = 587)		0,91 (0,71; 1,15); *p* = 0,42
*Dienstjahre*		
< 5 Jahre (*n* = 1068)	52,09; *p* ≤ 0,001	Referenz
5–< 10 Jahre (*n* = 505)		*1,40 (1,04; 1,89); p* *=* *0,025*
10 bis < 15 Jahre (*n* = 208)		1,44 (0,88; 2,36); *p* = 0,152
15 bis < 20 Jahre (*n* = 124)		1,77 (0,98; 3,22); *p* = 0,061
≥ 20 Jahre (*n* = 243)		1,05 (0,58; 1,91); *p* = 0,873
*BMI*		
< 18,5 (*n* = 38)	20,59; *p* ≤ 0,001	1,28 (0,60; 2,72); *p* = 0,524
18,5–< 25 (*n* = 997)		Referenz
> 25–< 30 (*n* = 755)		1,05 (0,83; 1,32); *p* = 0,688
> 30 (*n* = 497)		1,27 (0,96; 1,69); *p* = 0,095
*Beschwerden im Nacken oder sonstige chronische Beschwerden an der Halswirbelsäule*^*c*^		
Nein (*n* = 1543)	66,12; *p* ≤ 0,001	*1,54 (1,21; 1,95); p* *≤* *0,001*
Ja (*n* = 755)		*1,54 (1,21; 1,95); p* *≤* *0,001*
*Beschwerden im unteren Rücken oder sonstige chronische Rückenleiden*^*c*^		
Nein (*n* = 1354)	78,30; *p* ≤ 0,001	Referenz
Ja (*n* = 944)		*1,55 (1,24; 1,94); p* *≤* *0,001*
*Diabetes ohne Schwangerschaftsdiabetes*^*c*^		
Nein (*n* = 2258)	4,07; *p* = 0,044	Referenz
Ja (*n* = 40)		1,36 (0,59; 3,14); *p* = 0,47
*Asthma, einschließlich allergischem Asthma*^*c*^		
Nein (*n* = 2085)	14,84; *p* ≤ 0,001	Referenz
Ja (*n* = 213)		*1,62 (1,13; 2,33); p* *=* *0,009*
*Allergien ohne allergisches Asthma*^*c*^		
Nein (*n* = 1547)	19,91; *p* ≤ 0,001	Referenz
Ja (*n* = 751)		1,24 (1,00; 1,54); *p* = 0,051
*Arthrose*^*c*^		
Nein (*n* = 2211)	20,32; *p* ≤ 0,001	Referenz
Ja (*n* = 87)		*2,06 (1,07; 3,95); p* *=* *0,031*
*Hypertonie*^*c*^		
Nein (*n* = 2034)	18,13; *p* ≤ 0,001	Referenz
Ja (*n* = 264)		1,07 (0,76; 1,50); *p* = 0,709
*Depression*^*c*^		
Nein(*n* = 1981)	29,97; *p* ≤ 0,001	Referenz
Ja (*n* = 317)		*1,71 (1,26; 2,33); ≤* *0,001*
*Hypercholesterinämie*^*c*^		
Nein (*n* = 2117)	22,98; *p* ≤ 0,001	Referenz
Ja (*n* = 181)		1,29 (0,85; 1,95); *p* = 0,228

^a^ In die multivariable Analyse eingeschlossen wurden alle Variablen mit *p* ≤ 0,1 in den univariablen Analysen sowie das Geschlecht der Teilnehmenden

^b^ Wurden nicht separat in die Analyse eingeschlossen, da *n* < 10

^*c*^ In den letzten 12 Monaten

Mit einer erhöhten Chance für eine AU in den letzten 12 Monaten waren die tatsächliche wöchentliche Arbeitszeit (je Stunde Anstieg OR: 1,01 [95 % KI 1,0–1,02]; *p* = 0,003) und 5 bis unter 10 Jahre Beschäftigung im Rettungsdienst (OR: 1,40 [95 % KI 1,04–1,89]; *p* = 0,025) im Vergleich zu weniger als 5 Jahren assoziiert. Das Vorliegen einer der folgenden Krankheiten in den letzten 12 Monaten standen ebenfalls signifikant mit einer erhöhten Chance für eine AU im selben Zeitraum in Verbindung: Beschwerden im Nacken (OR: 1,54 [95 % KI 1,21–1,95]; *p* ≤ 0,001), (chronische) Beschwerden im (unteren) Rücken (OR: 1,55 [95 % KI 1,24–1,94]; *p* ≤ 0,001), Asthma (einschließlich allergischem Asthma; OR: 1,62 [95 % KI 1,13–2,33]; *p* = 0,009), Arthrose (OR: 2,06 [95 % KI 1,07–3,95]; *p* = 0,031) sowie Depression (OR: 1,71 [95 % KI 1,26–2,33]; *p* ≤ 0,001).

Alle Details zu den univariablen und multivariablen Analysen sind in Tab. 2 abgebildet.

## Diskussion

Diese Analyse zeigt, dass über 59 % der teilnehmenden Rettungskräfte in den vorangegangen 12 Monaten mindestens eine Arbeitsunfähigkeit hatten und dass diese mit dem Schulabschluss, dem Arbeitsumfeld, der wöchentlichen Arbeitszeit, den Dienstjahren sowie dem Vorliegen bestimmter (chronischer) Erkrankungen assoziiert war.

Laut der Berichterstattung der Techniker Krankenkasse war die durchschnittliche erkrankungsbedingte Fehlzeit im Jahr 2019 bei 15,1 Tagen je Erwerbsperson [24]. Bei den teilnehmenden Rettungskräften mit mindestens einer AU in den letzten 12 Monaten lag die durchschnittliche Anzahl der AU-Tage bei 19,4 Tagen sowie bei 11,4 Tagen für die Gesamtstichprobe. Daten der Universität Berlin zum Gesundheitsmonitoring von in Berlin tätigen Polizist*innen konnte zeigen, dass die Beamt*innen durchschnittlich 21 Tage in den letzten 12 Monaten, wegen einer Arbeitsunfähigkeit, nicht zur Arbeit gehen konnten [28].

Aktuelle Daten weisen darauf hin, dass ältere Mitarbeitende in der Wirtschaft nicht häufiger krank sind als ihre jüngeren Kolleg*innen [10]. In der vorliegenden Analyse war der Anteil der älteren Teilnehmenden (30–44 Jahre) mit mindestens einer AU in den letzten 12 Monaten vergleichbar mit der Altersgruppe der 45- bis 64-Jährigen und numerisch höher als bei den jüngeren Rettungskräften (18–29 Jahre). (Abb. 1b). Mit dem zunehmenden Alter nahm auch der Anteil der Teilnehmenden mit mehr als 30 AU-Tagen zu (Abb. 1f). Es ist bekannt, dass Arbeitnehmer*innen mit mehr als 30 AU-Tagen am Stück eine Lohnfortzahlung beanspruchen müssen. Des Weiteren muss der Arbeitgeber für gewöhnlich 30 Fehltage pro Jahr hinnehmen. Die vorliegende Analyse kann allerdings nicht darlegen, ob die 30 Tage zusammenhängend genommen werden mussten. Allerdings muss letztlich auch gesagt werden, dass in der multivariablen Analyse keine signifikante Assoziation zwischen dem Alter und einer AU in den letzten 12 Monaten identifiziert werden konnte (Tab. 2).

Erwähnt werden sollte, dass Teilnehmende zwischen 5 und 10 Jahren im Rettungsdienst eine höhere Chance für eine AU im Vergleich zur Referenzgruppe mit unter 5 Dienstjahren aufwiesen. Dieser Trend, wenn auch nicht signifikant und mit Ausnahme der Teilnehmenden mit > 20 Dienstjahren, ist auch für Teilnehmende mit 5 bis 20 Jahren Berufserfahrung erkennbar. Eine Arbeit von Heringshausen et al. (2010) zeigte, dass Rettungskräfte mit mehr Berufsjahren (≥ 4 Jahre) eine schlechtere Arbeitsfähigkeit sowie einen schlechteren allgemeinen Gesundheitszustand haben als Rettungskräfte mit weniger Dienstjahren [5]. In einer Studie zur Berufstreue von angehenden Rettungskräften wurde dargelegt, dass etwa die Hälfte der Teilnehmenden nicht länger als 10 Jahre im Beruf arbeiten will, und als Gründe dafür wurden neben einer fehlenden Rechtssicherheit im Beruf unter anderem die mentale und besonders die körperliche Belastung angegeben [6]. Wir vermuten, dass mit steigenden Dienstjahren die Belastung und somit die gesundheitlichen Probleme der Teilnehmenden zunehmen und so die Chance von Arbeitsunfähigkeiten prinzipiell ansteigt. Aufgrund der Daten von Hofmann und Macke (2020) [6] vermuten wir allerdings auch, dass die besonders resilienten Rettungskräfte im Beruf verbleiben und so die Chance für eine Arbeitsunfähigkeit bei denjenigen mit über 20 Dienstjahren wiederum mit denen mit weniger als 5 Jahren wiederum vergleichbar ist.

Der Bildungsabschluss erwies sich in der vorliegenden Analyse als assoziierter Faktor für eine Arbeitsunfähigkeit in den letzten 12 Monaten bei den teilnehmenden Rettungskräften. Zu ähnlichen Ergebnissen kamen auch Meyer et al. im Fehlzeiten-Report in der deutschen Wirtschaft 2020. Sie sahen dies unter anderem darin begründet, dass zumindest Akademiker*innen bessere Aufstiegschancen, größere Handlungsspielräume und ein höheres Gehalt haben. Dies stärkt die Identifikation mit dem eigenen Beruf und fördert die Motivation [10]. Die Arbeit im Rettungsdienst setzt keine akademische Laufbahn voraus. Dennoch ist zu vermuten, dass sich viele Kolleg*innen während ihres rettungsdienstlichen Arbeitslebens akademisch weiter qualifizieren möchten. Annahme hierfür bietet auch die Befragungsstudie von Hofmann & Macke (2020), in der unter anderem fehlende Aufstiegs- und Weiterbildungsmöglichkeiten als Gründe für einen beruflichen Wechsel angegeben wurden [6]. Zudem könnte es sein, dass Mitarbeitende während ihrer beruflichen Tätigkeit bereits studieren und nach ihrem akademischen Abschluss nicht im Rettungsdienst verbleiben. Die Tatsache, dass ein Großteil der teilnehmenden Rettungskräfte das Abitur als Bildungsabschluss angegeben haben, unterstützt diese Vermutung (Tab. 2). Dies könnte den Vergleich zur allgemeinen deutschen Wirtschaft sicherlich möglich machen [10].

Die Arbeitsumgebung bietet einen interessanten Einblick und ist im großstädtischen Gebiet im Vergleich zur städtischen und ländlichen Umgebung signifikant mit der erhöhten Chance für Arbeitsunfähigkeit assoziiert. Den Einsatzort betreffend stellt eine Großstadt mit mehrstöckigen Gebäuden, belebten Stadtplätzen und dichter besiedelten Klinikstrukturen tendenziell andere Herausforderungen an die Rettungskräfte als eine Kleinstadt oder eine ländliche Region. Eine mögliche Erklärung könnte sein, dass in einer Großstadt ein höheres Einsatzaufkommen pro Fahrzeug im Vergleich zum Land vorherrscht. Allein die Stadt Köln verzeichnete im Jahr 2020 172.855 Einsätze, an denen ein Rettungsmittel zum Einsatz entsendet wurde [3]. Dieses höhere Einsatzaufkommen kann sicherlich auch die Einsatzfrequenz für die einzelnen Rettungskräfte erhöhen, was sich negativ auf die Gesundheit auswirken könnte. So zeigten auch Heringshausen et al. (2010), dass Rettungskräfte mit mehr Einsätzen (≥ 4/8 h) eine schlechtere Arbeitsfähigkeit und eine höhere Burnout-Symptomatik aufwiesen als diejenigen mit weniger Einsätzen [5]. Eine Untersuchung an 276 Rettungskräften aus Deutschland konnte bei 44,3 % der Berufsfeuerwehrleute und bei 20 % der Mitarbeitenden von Hilfsorganisationen risikobehaftete, arbeitsbezogene Verhaltens- und Erlebnismuster nachweisen [29]. Dieser Zusammenhang zwischen Arbeitsbelastung und Gesundheit wird auch durch die in der vorliegenden Arbeit identifizierten Assoziation zwischen wöchentlicher Arbeitszeit und einer Arbeitsunfähigkeit in den letzten 12 Monaten angedeutet (Tab. 2).

Ein hohes Fahrtenaufkommen kann tendenziell für eine höhere physische und psychische Arbeitsbelastung auf den Rettungswachen sorgen. Es ist bekannt, dass das berufs- und arbeitsbezogene Stressempfinden als psychosozialer Risikofaktor für chronische Rückenschmerzen gilt [17]. Auch das Risiko für eine Depression steigt, je höher die Arbeitsintensität ist [16]. Basierend auf den Daten der vorliegenden Studie waren Beschwerden im Rücken und Nacken sowie das Vorliegen einer Depression in den letzten 12 Monaten mit einer erhöhten Chance für eine Arbeitsunfähigkeit im selben Zeitraum assoziiert (Tab. 2). Zu erwähnen ist noch, dass bei Berufstätigen in Pflegeberufen psychische und Verhaltensstörungen sowie Krankheiten des Muskel-Skelett-Systems und des Bindegewebes die häufigsten ICD-10-Kodierungen im Zusammenhang mit AU-Tagen sind [25]. Vielerorts wird zumindest versucht, der Belastung im präklinischen Rettungsdienst mithilfe von elektronischen Trage- und Transporteinrichtungen entgegenzuwirken, um somit die Belastung der Rettungskräfte zu reduzieren. Dies könnte in den folgenden Jahren zu veränderten Ergebnissen führen.

Auch Arthrose und Asthma sind in der vorliegenden Studie mit einer erhöhten Chance für Arbeitsunfähigkeit in den letzten 12 Monaten assoziiert. Von beiden Erkrankungen ist auch bekannt, dass diese in einzelnen Alters- und Geschlechtsgruppen bei Rettungskräften häufiger vorkommen als in der deutschen Allgemeinbevölkerung [11]. Daten aus Polen und den USA zeigen, dass Asthma zu 6,4 Krankheitstagen pro Patient*in bzw. Asthmaanfälle im Schnitt zu 5,4 Tagen Krankenstand führen [23].

Wie viele Krankentage durch Asthma oder andere Faktoren entstehen, welche in der vorliegenden Analyse mit einer Arbeitsunfähigkeit in den letzten 12 Monaten assoziiert sind, kann anhand der Studiendaten nicht festgestellt werden. Im Rahmen der vorliegenden Arbeit wurden nur die Krankentage in den letzten 12 Monaten insgesamt abgefragt und nicht, wie viele Tage durch bestimmte Faktoren aufgetreten sein können. Aufgrund der vorliegenden Ergebnisse wäre es sicherlich sinnvoll, in zukünftigen Studien expliziter den Einfluss der identifizierten Faktoren zu untersuchen, um ggf. präventive Maßnahmen entwickeln und ergreifen zu können.

Diese Analyse hat mehrere Limitationen. Erstens ist der Einfluss der Coronapandemie auf die Arbeitsunfähigkeit schwer einzuschätzen, da uns keine vergleichbaren Daten für den deutschen präklinischen Rettungsdienst bekannt sind, welche vor der Pandemie erhoben wurden und somit als Vergleich herangezogen werden könnten. So ist es aufgrund der Pandemie durchaus möglich, dass auch Kinderkrankheitstage/​‑quarantänen ggf. als AU-Tage mit erhoben wurden. Des Weiteren kann die Pandemie zu einem veränderten Fahrtenaufkommen geführt haben, und die Rettungskräfte stehen im häufigen und nahen Kontakt zu COVID-19-Erkrankten. Dies könnte das Anfallen von AU in dem Befragungszeitraum beeinflusst haben. Somit sollte diese Studie in einigen Jahren nach der Pandemie wiederholt werden. Zweitens sind die Angaben zur AU von den Rettungskräften selbstberichtet, sodass die Daten hier auf deren Ehrlichkeit und Erinnerungsvermögen beruhen. Des Weiteren wurde nicht nach AU-Tagen durch ärztliches Attest oder attestfreie Krankmeldungen, welche gewöhnlich für 2–3 Tage erfolgen können, unterschieden. Drittens lässt das Querschnittstudiendesign keine kausalen Interpretationen zwischen Ursachen für die Arbeitsunfähigkeit in den letzten 12 Monaten zu, sondern stellt Assoziationen zwischen der AU und den Merkmalen dar. Es wäre also zu empfehlen, eine vergleichbare Studie mit einem longitudinalen Studiendesign durchzuführen. Viertens konnte nicht nachvollzogen werden, in welchen Bundesländern und für welche Institutionen (Hilfsorganisationen/Berufsfeuerwehren/kommunale Trägerschaften) die Rettungskräfte tätig waren, was trotz zufälliger Auswahl der Studienteilnehmenden ggf. zu einem Bias bei der Auswahl der Studienteilnehmenden geführt haben könnte. Fünftens wurde im Fragebogen ausschließlich die AU und die Anzahl von AU in den letzten 12 Monaten abgefragt. Nicht ermittelt wurden Ursachen für eine AU.

## Fazit für die Praxis

- Diese Analyse konnte zeigen, dass das Arbeitsumfeld, die wöchentlichen Arbeitsstunden, der Schulabschluss, die Dienstjahre sowie bestimmte Erkrankungen mit einer Arbeitsunfähigkeit in den letzten 12 Monaten bei deutschen Rettungskräften assoziiert waren.
- Durch weitere Analysen dieser Art sowie longitudinale Studien zu den o. g. Faktoren und deren konkreten Einfluss auf den Gesundheitszustand könnten weitere Daten erhoben werden, um Ursachen und Folgen von Arbeitsunfähigkeit bei deutschen Rettungskräften besser zu verstehen.
- Es ist zu vermuten, dass basierend auf den identifizierten, assoziierten Faktoren, wie Rückenschmerzen, Depression oder auch Dienstjahren sowie der diskutierten Literatur, die Arbeitsbelastung im Rettungsdienst einen Einfluss auf die Arbeitsunfähigkeit haben könnte, und hier ggf. erste präventive Maßnahmen entwickelt werden könnten.
- Da insbesondere auch im Rettungsdienst ein Mangel an entsprechenden Fachkräften die Versorgungssituation verschärfen könnte, muss die Arbeitsfähigkeit von Rettungskräften bestmöglich erhalten werden.

## References

[1] Boudreaux E, Mandry C, Brantley PJ (1997). Stress, job satisfaction, coping, and psychological distress among emergency medical technicians. Prehosp Disaster Med.

[2] DESTATIS Qualität der Arbeit – Wöchentliche Arbeitszeit. https://www.destatis.de/DE/Themen/Arbeit/Arbeitsmarkt/Qualitaet-Arbeit/Dimension-3/woechentliche-arbeitszeitl.html. Zugegriffen: 23. August 2022

[3] Feuerwehr Köln Jahresbericht 2020.

[4] Hering T, Beerlage I (2004). Arbeitsbedingungen, Belastungen und Burnout im Rettungsdienst. Notfall Rettungsmed.

[5] Heringshausen G, Nübling M, Brauchle G (2010). Arbeitsplatz Rettungsdienst – Arbeitsfähigkeit als Indikator für Arbeitsbedingungen im Rettungsdienst. Zentralbl Arbeitsmed Arbeitsschutz Ergon.

[6] Hofmann T, Macke M (2020). Berufstreue von angehenden Notfallsanitäter*innen – Eine Befragung von Auszibildenden über ihren Berufsverbleib.

[7] JASP Team (2021). JASP (Version 0.16).

[8] Kim J, Lee N, Kim JY (2019). Organizational response to workplace violence, and its association with depressive symptoms: a nationwide survey of 1966 Korean EMS providers. J Occup Health.

[9] Leszczyński P, Panczyk M, Podgórski M (2019). Determinants of occupational burnout among employees of the Emergency Medical Services in Poland. Ann Agric Environ Med.

[10] Meyer M, Wing L, Schenkel A, Meschede M (2021). Krankheitsbedingte Fehlzeiten in der deutschen Wirtschaft im Jahr 2020. Fehlzeiten-Report 2021.

[11] Möckel L, Arnold C, May T, Hofmann T (2022). The prevalence of diseases in German emergency medical services staff: A survey study. Arch Environ Occup Health.

[12] Möckel L, Gerhard A, Mohr M (2021). Prevalence of pain, analgesic self-medication and mental health in German pre-hospital emergency medical service personnel: a nationwide survey pilot-study. Int Arch Occup Environ Health.

[13] Mohr M, Schillings J, Möckel C (2021). Mit Schmerzen und der Schmerzmitteleinnahme assoziierte Faktoren bei deutschen Rettungskräften: Eine Post-hoc-Analyse. Arbeitsmed. Sozialmed. Umweltmed..

[14] Olschowka N, Möckel L (2021). Aggression and violence against paramedics and the impact on mental health: A survey study. J Emerg Med Trauma Acute Care.

[15] Rademaker M (2019). NotfallsanitäterInnen im Einsatz – Immer auf dem Sprung. Mag Beamtinnen Beamte.

[16] Rau R, Henkel D (2013). Zusammenhang von Arbeitsbelastungen und psychischen Erkrankungen. Nervenarzt.

[17] Raspe H, RKI (2012). Rückenschmerzen.

[18] Robert Koch-Institut (2017). Fragebogen zur Studie „Gesundheit in Deutschland aktuell“: GEDA2014/2015-EHIS. J Health Monit.

[19] Sahebi A, Jahangiri K, Sohrabizadeh S, Golitaleb M (2019). Prevalence of workplace violence types against personnel of emergency medical services in Iran: a systematic review and meta-analysis. Iran J Psychiatry.

[20] Schmitt L (2018). Betriebliches Gesundheitsmanagement im Rettungsdienst – Ein Muss. Herausforderung Notfallmedizin.

[21] Sefrin P, Händlmeyer A, Stadler T, Kast W (2021). Erfahrungen zur Gewalt gegen Rettungskräfte – aus der Sicht des DRK. Notarzt.

[22] Sieber F, Kotulla R, Urban B (2020). Entwicklung der Frequenz und des Spektrums von Rettungsdiensteinsätzen in Deutschland. Notfall Rettungsmed.

[23] Stróżek J, Samoliński B, Kłak A (2019). The indirect costs of allergic diseases. Int J Occup Med Environ Health.

[24] Techniker Krankenkasse (2020). Arbeitsunfähigkeit.

[25] Techniker Krankenkasse (2019). Gesundheitsreport 2019 – Pflegefall Pflegebranche? So geht’s Deutschlands Pflegekräften.

[26] Lehweß-Litzmann R, Hofmann T (2022) Fachkräftenachwuchs für den Rettungsdienst? Wie auszubildende Notfallsanitäter:innen ihre berufliche Zukunft sehen. SOFI Working Paper 2022-24. https://sofi.uni-goettingen.de/fileadmin/user_upload/WorkingPaper_Lehwess-Litzmann_Hofmann_2022.pdf. Zugegriffen: 23. August 2022

[27] (2022) Gesetz über den Rettungsdienst sowie die Notfallrettung und den Krankentransport durch Unternehmer (Rettungsgesetz NRW – RettG NRW). https://recht.nrw.de/lmi/owa/br_text_anzeigen?v_id=10000000000000000325. Zugegriffen: 18. August 2022

[28] Kleiber D, Rotter M, Stark S, Renneberg B (2014) Gesundheitsmonitoring in der Polizeidirektion A & Risikokonstellationen für die vorzeitige Versetzung in den Ruhestand. https://www.ewi-psy.fu-berlin.de/einrichtungen/arbeitsbereiche/klinische_psychotherapie/Forschung/Drittmittelgefoerderte-Projekte/Abgeschlossene-Projekte/Polizei-Projekt/Gesundheitsmonitoring-2013.pdf. Zugegriffen: 17. August 2022

[29] Thielmann B, Böckelmann I, Schumann H (2022). Work-related behavior and experience patterns among ambulance service personnel of different organizational structures in urban and rural regions. J Occup Environ Med.

